# Nonpharmacological Compensation of Aggressive Behavior of Individuals with Moderate Intellectual Disability and Behavioral Disorders—A Case Study

**DOI:** 10.3390/ijerph19159116

**Published:** 2022-07-26

**Authors:** Michal Vostrý, Barbora Lanková, Ilona Pešatová, Otakar Fleischmann, Jaroslava Jelínková

**Affiliations:** 1 Research Center, Faculty of Health Studies, University J. E. Purkyně in Ústí nad Labem, 40096 Ústí nad Labem, Czech Republic; 2 Department of Special and Social Education, Faculty of Education, University J. E. Purkyně, Ústí nad Labem in Ústí nad Labem, 40096 Ústí nad Labem, Czech Republic; ilona.pesatova@ujep.cz; 3 Department of Primary and Pre-Primary Education, Faculty of Education, University J. E. Purkyně in Ústí nad Labem, 40001 Ústí nad Labem, Czech Republic; barbora.lankova@ujep.cz; 4 Department of Psychology, Faculty of Education, University J. E. Purkyně in Ústí nad Labem, 40096 Ústí nad Labem, Czech Republic; otakar.fleischmann@ujep.cz; 5 Department of Languages, Faculty of Education, University J. E. Purkyně in Ústí nad Labem, 40096 Ústí nad Labem, Czech Republic; jaroslava.jelinkova@ujep.cz

**Keywords:** aggressive behavior, intellectual disability, rehabilitation, special education, psychology

## Abstract

The article discusses issues associated with the manifestations of aggressive behavior in an individual diagnosed with moderate intellectual disability and behavioral disorders (according to ICD-10; F7; F711—moderate intellectual disability, significant impairment of behavior requiring attention or treatment). In the research survey, we focused on a client corresponding with relevant features. The research was carried out at the beginning of hospitalization, ongoing hospitalization, and the end of hospitalization, followed by a recommendation to limit the legal capacity of the client and his placement in a residential care home. The case study points out individual approaches to special education and psychology and outlines the key steps in the cooperation of selected helping professions suggesting conclusions and recommendations for practice regarding these selected issues. Upon the termination of our investigation, there was a rapid deterioration of the client being admitted to the intensive care unit with a follow-up treatment in a long-term intensive care unit.

## 1. Introduction

Intellectual disability, formerly known as mental disability, is a condition characterized by marked below-average intellectual functioning. It is accompanied by an adaptive behavior disorder that manifests before affected individuals reach the age of 18. Mild, moderate, severe, and profound levels are classified according to ICD-10. Furthermore, according to ICD F70-F79, intellectual disability is a state of stopped or incomplete mental development, which is characterized by impaired skills, manifested during the developmental period, and affecting all components of intelligence, i.e., cognitive, speech, motor, and social abilities. Intellectual ability and social adaptability are subject to a change over time, and even lower values of intellect can be improved by exercise and rehabilitation. The degree of intellectual disability is usually measured by standardized intelligence tests determining the IQ cutoffs for moderate levels 35 to 49 (which corresponds to a mental age of 6 to 9 years in adults) resulting in a developmental delay in childhood. Nevertheless, some individuals can develop a certain level of independence and self-sufficiency, achieving adequate communication and school skills. Adults will need varying degrees of support at work and within society [1,2]. Due to the growing number of people suffering from intellectual disabilities, it is becoming a social problem. According to meta-analyses, about 1% of the population globally suffers from an intellectual disability [3,4,5], which is associated with multicausal risk factors including, e.g., genetic, nongenetic, or acquired causes. However, in rare cases, the etiology itself is unknown. Genetic factors, i.e., chromosomal abnormalities, inherited genetic traits, and single-gene disorders, are responsible for 30% to 50% of the cases. Other ontogenetic causes include prenatal, perinatal, postnatal, and environmental factors. Maternal conditions such as asthma, diabetes, hypertension, and epilepsy are prioritized [6,7]. Other factors include the use of tobacco, alcohol, and other addictive substances as well as the older age of parents and the mother’s low education.

Postnatal factors include infections, exposure to toxic substances, developmental disorders, central nervous system malignancy, or chronic malnutrition. Associated factors interplay with environmental factors and sociodemographic and socioeconomic characteristics of the population [8,9,10]. In general, physicians speak of a developmental disability manifested in infancy or early childhood. In some cases, the disability cannot be diagnosed until the individual is older than ~5 years of age when standardized measures become more reliable [11,12].

### Background

Aggressive behavior ranks among significant problems associated with individuals with intellectual disabilities. Global estimates of the prevalence of aggressive behavior range from 9% to 23% [13]. According to more recent research, the estimates vary with authors reporting a prevalence of 5% to 15% [14,15,16,17,18,19]. However, the etiology of problem behavior has not been fully understood. It probably consists of several interacting personal factors, e.g., a lifestyle, and mental and physical health promotion [14,15,20]. Aggressive behavior can be caused, among other things, by poor anger control or limited anger management skills. It is in the absence of self-regulation of emotional responses that innocuous environmental stimuli can create disproportionate emotional arousal eventually resulting in aggressive behavior [21,22]. In the case of aggressive behavior, physicians often encounter verbal and physical aggression. Physical aggression in the form of self-aggression or hetero aggression often results in damage to the health of others or the aggressor. According to studies, even mild aggression can adversely affect an individual diagnosed with an intellectual disability and his or her family leading to isolation and/or stigmatization [23]. Aggressive behavior has a negative impact on the human environment as well as on the aggressor consequently also interfering with social activities. Furthermore, there is considerable risk of physical injuries. Caregivers as well as family members may suffer from negative emotions, stress, and fear of physical assault over an extended period. Selected research mentions that aggressive behavior tends to persist over time. In that case, individuals may exhibit more than one form of aggressive behavior at the same time, e.g., physical, verbal, heteroaggressive, or self-aggressive behavior. Factors associated with such behaviors include psychiatric disorders, low levels of intellectual function, gender, and genetic syndromes. We must not forget about environmental factors such as negative interactions between employees or the desire to escape everyday tasks [23,24,25,26,27].

Intellectual disability can occur without or concurrently with other somatic or mental disorders and the level is usually measured by standardized intelligence tests that can be replaced by scales determining the degree of social adaptation in a given environment. Such scale measurements can determine only approximately the degree of intellectual disability. A diagnosis delivered by a qualified diagnostician based on general intellectual functions corresponds to the current state of the client’s mental functions.

## 2. Case Study

To protect personal data, we present only basic, crucial, and anonymized information obtained from the analysis of medical records and partial interviews with the permission of legal representatives in anonymized form omitting unnecessarily detailed description (e.g., the relevant place of hospitalization). Specifically, we drew the necessary information from the progress and discharge report with the client’s consent. We present it in the form of a case report with the permission of legal representatives in an anonymized form.

The client (17.8 years of age; during the intervention 18.0 years of age) was hospitalized for a long time (since December 2020), in the care of a child and adolescent psychiatry outpatient clinic with the diagnosis of moderate intellectual disability accompanied by severe behavioral disorders and stuttering requiring treatment. Muscle hypotension and microcephaly were evident from birth. Psychomotor disability and atopic eczema from infancy. Hyperkinetic syndrome and obesity have developed and are worsening. From the age of 13, scoliosis worsened into the gibbus itself. From the age of 14, aggressive behavior appeared, especially in a group, and at present also progressively in the home environment. The signs of CNS dysfunction dominate the clinical picture. Uncoordinated movements are evident in gross and fine motor skills. Overall, the client is motor-clumsy. Attention is short-lived and increased mental fatigue and stuttering are evident in speech. There is emotional instability, along with reduced frustrated tolerance and increased readiness for affective behavior accompanied by aggressive outbursts. There is a tendency to negativism and increased resistance to people near him. The client reacts anxiously under stress, his reactions are unexpected and correspond to a younger child. Overall nonindependent, the subject requires an individual approach with consistent supervision and assistance. Educated according to the Framework Education Programme (FEP) for Elementary Education—part I (in the Czech Republic it is FEP for special primary schools). He was repeatedly expelled from school due to aggressive behavior and noncooperation.

He attended a special primary school for nine years, which he left. He does not go to school at the moment. (He attended a special primary school for 2 years, then transferred to another special primary school where he studied for 7 years.) He completed compulsory schooling but did not acquire the basics of education. Due to motor clumsiness and instability, assistance is required in routine tasks (getting dressed, putting on shoes, undressing, etc.). Regarding eating the client is able to feed himself under supervision, which is necessary mainly to prevent overeating causing his obesity. The food must be ready, and the eating is unclean. He is not able to orient himself in time, he does not understand clocks, and he needs to be accompanied in an unknown environment. He is easily influenced, which increases the risk of exploitation. His comprehension is minimal, he is not able to make independent decisions, and he is not able to plan or organize his daily routines. He is completely nonindependent in personal hygiene and frequently manipulates the rectum. Medication is necessary in case of aggression and must be administered by an adult and another person. Drug attenuation is evident after medication.

Since May 2021, he has been in the care of a psychiatric outpatient clinic with a permanent behavioral disorder. He is accompanied by a legal representative there. He behaved defiantly with rage. According to the legal representative, manifestations of the behavioral disorder have worsened in recent weeks and the legal representative is no longer able to handle the client. He demolished their apartment and physically attacked the occupants of the house, and the police intervened at the place of residence. After their departure, the same manifestation of behavior was repeated; therefore, he was placed in the psychiatric ward of the hospital. He was subsequently released. Objectively, the client is lucid, passively cooperating, and restless. Thinking is coherent and simple. Emotionally unstable with a behavioral disorder. Hospitalization in a psychiatric hospital is recommended to stabilize his condition. He is unmanageable at home. Recent medications used by the client: Tisercin 25 mg (2-0-2), Restigulin 15 mg (1-0-1), Sertraline 50 mg (2-0-0), Rivotril 0.5 mg (2-2-2), and potassium chlorate (1-0-0), which has a subduing effect on the client. Many of the meds are administered in the morning and the evening, so the appropriate time of therapy was determined sometime during the day.

**Summary:** recommended permanent support and care, granting of a disability support pension of the third level, the proceedings to limit the client’s legal capacity according to the valid legislation in the Czech Republic was commenced. In the case of ACC, the patient was educated on the VOKS (interchangeable picture communication system—widespread in the Czech Republic). A plan was created so that the same system would be used in the home and school environment.

## 3. Therapy Design

### 3.1. Special Education

The design of the therapy is based on the knowledge that the boy completed compulsory schooling and that the education took place according to the FEP for Elementary Education—part I (in the Czech Republic it is FEP for special primary schools). Given the boy’s age, it is important to continue his education so that his skills, abilities, and habits develop. It is advisable to continue education at an elementary school for pupils with mild intellectual disability. This is a one-year study with the possibility of a prolongation, as the scope and severity of the boy’s special educational needs entitle him to further education in the special education regime.

For schooling at the practical school, it is necessary to develop an individual educational plan with structured teaching reflecting the boy’s emotional instability, behavioral disorders, impaired communication skills, and perception of time sequence, etc. Furthermore, it is necessary to respect the need for a longer time to acquire knowledge and practice partial skills. First, affective behavior must be rigorously monitored in the family and at school. Second, negativity, defiance, and aggressive outbursts must be ruled out as those that can harm family members and classmates (due to the proportions and strength of the boy). Overall, intensive cooperation with the school and school counselling facility are necessary. The counselor, most often a special educator, analyzes and evaluates possible causes and manifestations of the boy’s behavior and focuses on educational effects and therapeutic procedures that might help to improve cooperation with the boy at school and in the home environment. We also recommend using an individualized motivation system based on the specific interests of the boy and selected educational activities and leading the client to maintain a certain order and regime at school and home. In this way, the stress load should be alleviated, and the boy would have a greater sense of security and the ability to anticipate the consequences. It should also calm the boy in terms of his problem behavior and strengthen his self-regulation skills. The boy needs clear rules at school and home. Clarity at school as well as home with regard to rules will help him to better orientate himself not only in terms of activities during schooling but also in terms of school regime and time organization, i.e., local, spatial, and temporal structuring. The boy’s daily schedule needs to be structured visually using cards or workbooks for the daily and later weekly regime and followed consistently with patience both at school and at home (family members need to be given training).

Due to the limited ability to communicate, it is appropriate to regulate the boy’s experience using process diagrams with emotions and cards that express individual emotions and help to recognize and express his experience and feelings. From the point of view of the boy’s deficit communication, it is necessary to use alternative and augmentative communication systems (AAC) (Regarding AAC, the patient was educated with the use of VOKS (visual communication system), which is widespread in the Czech Republic. A plan was created so that the same system would be used in the home and school environment), e.g., in the form of simple pictograms, or communication books with specific pictures for a specific area. It is also appropriate to create a simplified portfolio of schoolwork and build on the boy’s current level of symbolization so that the boy understands the image. The weakened receptive component of the boy’s speech is characterized by a reduced understanding of the statements of others, which leads to the need to simplify the message, connect with his opinion, and deliver instructions step by step. To develop communication and appropriate responses, it is possible to use, for example, social reading or so-called three-component educational cards designed to develop passive and active vocabulary, understand the content of terms and words, and create connections between them. In a suitable way, these educational cards can also be used for the development of memory functions, as well as global reading. Devices, so-called communicators with voice output, can also be used for speech production, which serves to support significantly impaired communication skills [28]. In communication with a boy who is developing stuttering, it is necessary to leave enough space for the formulation, rhythmize speech, melodize, and use well-established appropriately regulated communication stereotypes and not to hurry the formulation. In contact with the boy, try to keep a calm voice, give instructions in a simplified and understandable way, and do not rush the boy; instead, leave him time to process information. Furthermore, use the means of nonverbal communication—visual contact, touch, gestures, facial expressions, physical distance, etc.

In all educational activities, it is necessary to take into account the mental age of the boy: his increased mental fatigue, reduced frustrated tolerance, increased readiness for affective behavior, aggressive explosions, tendencies to negativism, and increased defiance. It is under stress that the boy’s reactions are unexpected, anxious, and aggressive. Impulsive and aggressive behavior resulting in a raptus of aggressive behavior can be prevented by intensive educational work with the school and family. The boy can be influenced and calmed down in a suitable form with various interventions, and one can easily communicate with him so that he understands the content of the information. Therapies and interventions should concentrate on coping with emotionally and socially difficult situations, e.g., selected intervention techniques [29], while practicing social skills and coping in stressful situations, such as traffic light techniques [30]. Furthermore, it is necessary to use relaxation techniques, breathing exercises, listening to relaxation music, etc. during education. Similar breathing exercises and relaxation techniques ought to be applied at home as well. It is also possible to use systemic music therapy [31], which can be implemented in various therapeutic situations—individual, group, active, or receptive. In our case, it is appropriate to use individual active music therapy to help the client to express emotions by playing a musical instrument. Furthermore, work with emotions focus on aggressive manifestations and gradually improve self-control. Music therapy includes handling the feelings of tension and inducing relaxation (through working with tempo, dynamics, rhythm, and joint improvisation). The whole process includes, e.g., an extensive area of therapeutic activities focusing on the development of social interactions and communication, managing self-expression, reducing problem behavior, working with emotions, calming, and relieving tension acceptably and safely. According to Müller [32], music enables the satisfaction of various needs of clients, i.e., physical, emotional, intellectual, and social, as it has nonverbal communicative, structural, emotional, and creative qualities. In terms of success, the above-mentioned instructions must be observed in a home setting. Therefore, it is necessary to conduct repeated training with the family so that the boy can communicate with people he knows well or less well in an adequate way and gradually improve his functional abilities. The family needs to be instructed in detail about the nature of the moderate intellectual disability and possible educational opportunities. It is essential to write an educational plan for the family and to help set the boundaries of good behavior.

It is necessary to cooperate in the form of regular therapies with the boy and his family, to focus on educational methods and methods of remuneration, and to determine adequate educational means of rewards and punishments [33]. Moreover, during therapies with the boy and his family, we work with emotions and find out the causes of individual aggressive manifestations (which the boy may also experience as a threat). We assume that targeted therapy will gradually correct the boy’s aggressive manifestations and emotional instability. The education of the boy and his family must also address the practice of hygiene habits and changes in eating habits (cleaner eating, not overeating) and embrace appropriate responses to the boy’s sexual manifestations while providing him with sexual intimacy.

### 3.2. Psychology Intervention

In the context of psychological intervention and possible psychotherapy, it is again necessary to consider the relatively significant cognitive deficit and insufficiency in the control of emotions and behavior. In the cognitive area, concerning the mental deficit, the client has a clear problem in understanding situational contexts and tends to evaluate them as threatening and frustrating, thus activating the experience of anger. It is anger, in addition to fear, that appears to be a frequent emotional reaction to a situation of danger.

As stated by Dentemarová and Kranzová [34], anger as a feeling of displeasure results from, e.g., injury, injustice, insult, or aversion. As a constructive emotion, anger signals that there is something wrong in our environment, something that could endanger us, which we consider an obstacle on the way to the goal. Anger as an energizing emotion gives us the energy and strength to deal with those obstacles. Anger can even protect us if we express it in the right way and at the right time. On the other hand, anger has a destructive aspect. When anger spirals out of control, behavior becomes unconstructive. People are physically or verbally aggressive, and the consequences of such behavior can be tragic. The intensity of anger depends primarily on the determination of anger and residual excitement. Anger is perceived as a combination of psychological arousal and the cognitive designation of this arousal. These cognitions are themselves influenced by internal and external factors and behavioral responses to the situation [35]. Both environment and imaginative stimuli can evoke anger [36]. Our client has a problem mapping both imaginative and real stimuli that can be experienced as threatening. However difficult it may be due to his reduced ability to understand and express himself, it is necessary to learn it.

Three determinants, i.e., cognitive processes (assessment, expectations, private speech, etc.), external events (e.g., frustrations, threats) and behavior, or reactions to behavior, are mainly involved in the actual activation of anger [37]. Higher brain centers in the frontal area provide control of our behavior and the coordination of other cognitive functions. The executive functions of the frontal lobes include impulse control, emotional control, cognitive flexibility, working memory, self-monitoring, planning and prioritization, initiation, organization, and coordination of mental and physical activity. The evaluation of executive functions is difficult because they coordinate individual functions, such as memory, language, and spatial orientation. Disruption of any of these functions can affect planning, organization, and self-control. If we want to influence inadequate behavior in the form of reactive (affective) aggression, which is considered nonpathological aggression, it is necessary to focus on the cognitive area and thus affect the emotional and behavioral areas. The prefrontal cortex allows you to better manage difficult situations and can eliminate negative experiential and physical components of emotions.

The brain lobes can be strengthened (as muscles are strengthened) by various exercises. Due to the huge neuroplasticity of our brain, we can “reprogram” the brain by creating new synapses (connections between neurons). The prefrontal cortex perfuses and develops when we actively focus on learning, planning or problem solving. In addition to the development of cognitive functions, the benefit will be the ability to better process negative emotions [38]. For training and strengthening of executive brain functions, it is appropriate to start, for example, with elements of rational-emotional therapy by A. Ellis ([39]; we respect the limits with the client’s diagnosis). It is a directive educational procedure based on the theory of the causes of emotional disorders, i.e., the ABC theory. According to Ellis (ibid.), emotional reactions, such as anger, fear, and dissatisfaction, which we experience (C), are not a direct consequence of a certain external event (A) but result from our opinions, which give a certain meaning to an event play a significant role here (B). Our behavior, therefore, depends on the emotions that are evoked by the importance we attach to a particular matter. Most irrational opinions need to be corrected by analysis, explanation, and clarification. However, cognitive work has its limits depending on the level of the individual’s mental level. It is advisable to divide the activity into several steps that will be performed together with the client creating a certain *protocol of anger*:

Step 1—What made me angry today?

Step 2—How did I react? (I screamed, I cried, I ran away, I beat someone else...)

Step 3—How do I want to react next time?

Step 4—Step training, i.e., I imagine the situation that upsets me, and I train better, positive, constructive reactions that I noticed in one step before. (I practice a deeper breath, I try to see the situation from a different perspective, etc.)

Step 5—Discuss *the protocol of anger* with a teacher or a parent, who should evaluate this work positively, advise the client, and stimulate him to continue.

The protocol of anger is, in essence, the mapping of the triggers of anger leading to undesirable behavior, to which the people affected then respond with negative feedback (punishment), which again provokes anger. So, this is a kind of vicious circle that needs to be broken. An appropriate tool to reduce unwanted behavior is operant conditioning, where the occurrence of *rewards* and *punishments* is adequately used. Instead of *punishment* for inadequate behavior (as long as it does not endanger the health or life of the individual or people near him), *rewards* for any desired behavior are preferred. We work with the assumption that the reward is accompanied by a pleasant and punishment by an unpleasant experience. Consequently, what we experience as pleasure, we tend to repeat and seek. Thus, if the desired behavior is associated with a pleasant experience, the individual tends to repeat it. If unwanted behavior is associated with an unpleasant experience, the individual tends to avoid it.

This approach is especially suitable for individuals with mental deficits, where we prefer an appeal to the emotional rather than the cognitive area. The system of intermittent reinforcement can shape actions such as dressing and eating and thus reduce erratic or aggressive manifestations of the individual. The well-thought-out procedure should be applied daily for 1–2 months. Reinforcement in the form of rewards is necessary, i.e., what the individual experiences as pleasing. Working with a system of rewards can result in the so-called voucher technique, which an individual obtains for reasonable expressions and in an exchange for a certain number of bonuses the client can use various predetermined benefits. For individuals with less severe mental deficits, it is also possible to recommend the technique of contracts, which take the form of a written agreement and relate to adverse behavior or bad habits. By adhering to the contracts, the individual will gain some preagreed benefits. These are training programs; the mentioned techniques are effective for improving self-control and regulation of one’s behavior. As far as the emotional area is concerned, it is necessary to consider the principle of constraining the affect of anger, which leads to the escalation of the so-called arousal. Increasing arousal leads to an increase in tension and thus to an increase in affective irritability. Thus, the individual tends to evaluate and experience the stimuli as negative, which again leads to an increase in internal tension. Therefore, it is necessary to apply controlled anger ventilation. Thus, we do not forbid the client to be angry; on the contrary, we support it in the use of so-called paradoxes, but we do so in the right place and at the right time. After the catharsis, we can then proceed to the mental processing of the causes of anger, i.e., the joint elaboration of the above-mentioned protocol of anger and the search for ways to respond adequately to the situation.

We consider it necessary to emphasize that even from a psychological point of view, it should always be a comprehensive approach, where the cooperation of all those involved in the care of individuals is necessary. The work is demanding not only due to the need for complexity but also consistency. Any inconsistency can mean a return to the original state.

### 3.3. Intervention Summary

The summary of approaches was limited by the health status of the patient. Therefore, we present the most accurate possible procedures that we wanted to apply. We also consider these procedures to be effective in managing aggressive behavior in patients with mental disabilities.

The combination of special pedagogy and psychology enabled us to set up approaches to managing permanent behavioral disorders (according to DSM-IV, repeated and permanent violation of the basic rights of others, or major social norms). In addition to the classical approaches that we mentioned above, we also focused on the use of elements of music therapy. Above all, we chose the form of creative music therapy according to J. Kantor. In this case, improvisation is primarily preferred. Various musical instruments and spontaneous expressions of the individual are used during improvisation. Through the regular application of music therapy, we wanted to reduce aggressive behavior. Before the patient’s condition worsened, we applied part of the intervention we were planning. The patient initially did not respond well to group therapy with other individuals. Thus, a primarily individual form was chosen. In the case of the scream, the classical music of nature and the usual means failed. We focused on the fact that if the patient’s behavior escalated and aggression increased, this also affected the music therapy itself. We played more dynamic, dramatic music. We accompanied this with reading, where we worked intensively with the tone of voice. The patient thus fixed aggressive behavior with this drama. However, with the subsequent dimming of music or reading, the patient calmed down from the beginning. There was relaxation itself.

In the method of rational emotional therapy (inspired by A. Ellis), we proceeded according to three main exaggerating ways of reacting (exaggeration, low ability to bear adverse circumstances, and devaluing oneself or other people). These three methods reflected our patient’s condition intensively. As part of the model activities, the patient learned to manage normal daily activities. One example is classic carbonation. The patient was used to being given food immediately whenever he asked for it. In the case of refusal, aggression increased to such a stage that he physically attacked staff or family, for example. Hospitalization was subsequently required. As a model activity for reflecting emotional therapy, we tried to reduce these impacts so that the patient realizes that possible rejection is not a reason for aggression. We realize that this therapy is aimed more at patients who realize that their life could have a completely different dimension. This awareness, however, is significantly limited in persons with mental retardation. That is why we proceeded to use the elements of this therapy and not the whole complex approach. It was important for us that these elements permeated deeply and helped to remove the emotional burden that had arisen. In this case, de facto, modeling and roleplaying were combined with problem solving.

We are therefore based on the fact that regular intervention and work with the patient can nonpharmacologically influence the aggressive manifestations of the patient. In our case, nonpharmacological means the creation of such activities that will dampen aggressive behavior in the long term. From the observations and interviews, it was evident that the patient was not handled professionally. The reason was the reluctance of the legal representative. Thus, the patient was primarily confined to the home environment. In this environment, he did not have enough stimuli aimed at the development of motor, cognitive, emotional, and other areas. Our goal was to create such conditions that would reflect the age and individual needs of the patient. Unfortunately, the patient’s state of health did not allow us to better verify these approaches. That is why we move more on a general level, where it is assumed that such an approach can be effective and help to manage aggressive behavior.

## 4. Discussion

Behavior that can become a potential source of danger to individuals or people around them places a burden on caregivers. Nevertheless, it also puts a burden on teachers, family members and everyone who encounters these manifestations. It is these manifestations that can disrupt the educational process together with the social adaptability of the individuals [40]. Based on professional sources, problem behavior can be considered a synonym for maladaptive and challenging behavior. Such behavior can be further defined as culturally abnormal and of such intensity that it can be threatening and limiting in daily life. Aggressive behavior can be manifested as aggression toward others or property along with self-harming behavior. Stereotypical and oppositional behavior often occurs. The risk of clinical levels of problem behavior is evident from a very early age, i.e., around 2–3 years of age. Individuals with intellectual disabilities have a lower rate of prosocial behavior, i.e., behavior that is responsible for increasing social competencies and successful social interaction along with establishing relationships with peers [41,42].

From a general point of view, behavioral problems can be common in individuals with intellectual disabilities. However, it depends on the nature and severity of the problem. Primarily, we encounter mild behavioral problems, while on the other hand, we encounter serious behavioral problems. Most problem behaviors are considered undesirable and inappropriate by society [43,44]. In such cases, preventive programs that can be used autonomously or as part of outpatient or residential services appear to be effective. The plan was also to apply an individualized motivational system of educational activities based on the boy’s interest (suggested in Section 3.1). By doing so, we would lead him to a certain order. Unfortunately, due to health reasons, this concept has not been considered, but we are aware that it would be one of the key approaches. It is necessary not to neglect the mental as well as often physical load that is directed to the family of the client. They often deal with stress and further developing mental health problems [45].

As part of comprehensive approaches, we point out the importance of working directly not only with a specific individual client who has been diagnosed with behavioral disorders but also with the family. It is the family that needs to be educated on how to deal with the situation effectively. The use of special pedagogical and psychological approaches seems to be effective for this purpose. These approaches focus on the needs of the people involved. Comprehensive approaches give rise to a multidisciplinary team, which focuses on the issue, and includes experts from the so-called helping professions [46].

## 5. Conclusions

The article reflects pedagogical and psychological approaches in the case study of a client with a moderate intellectual disability. The primary issue was the severe raptus of aggressive behavior. The family analysis identified a number of obvious reasons that could have exacerbated the situation. Such causes include primarily insufficient stimulation in the family environment and failure to meet the needs of the client. However, an insufficient number of stimuli may not be the primary source of such behavior; albeit, it may, to some extent, affect its intensity. The proposed therapies are focused not only on working with the client himself but also with family members. From the point of view of helping professionals, it is necessary to focus on a comprehensive approach and create suitable conditions for the development of a particular client. The design of the therapy reflects the individual characteristics of the selected client. We also planned to make further observations to find out to what extent the therapy design was successful.

Although during our cooperation some improvements in the monitored indicators were evident, we were not able to prove its retrospective verification as the client’s condition was worsening after having attacked his father resulting in a stab wound.

The attack was the reason for the client’s hospitalization in a psychiatric hospital and a pharmacotherapy. However, the situation continues to deteriorate. The client after being confined to bed is currently with bilateral pneumonia and COVID-19 infection. He also suffered an 18-min cardiac arrest, which further exacerbated his state. According to the latest information, the client’s condition is severe, without any response to any stimuli. He is now primarily in the care of doctors. From the point of view of helping professionals, approaches to basal stimulation can be applied.

Pupils with a certain type of mental disability are educated in the Czech Republic in accordance with the Education Act (Act No. 561/2004 Coll.), in § 48 of this law, it points out the possibilities of educating pupils with moderate and severe mental disabilities or with simultaneous disabilities with multiple defects and with autism. It is explicitly stated here that those with moderate mental disabilities are educated in a special elementary school, at the request of a legal representative. Education in these types of schools takes 10 years. The legislation makes it clear that if pupils are educated at a special primary school, they will acquire basic of education. This allows them to continue to one-year and two-year practical schools. However, this did not happen in our case.

We want to point out the rarer but more extreme manifestations of aggressive behavior, which can have a destructive impact not only on the client but also on the family itself. During our investigation, proceedings were initiated to limit the legal capacity under the applicable legislation in the Czech Republic (based on Act No. 89/2012 Coll., the Civil Code and Act No. 292/2013 Coll., on special court proceedings). Since, according to applicable laws, the client has reached the age of adulthood, he could not be placed primarily in a special facility where he would be provided with adequate care. However, the process was suspended due to his current critical state.

## Data Availability

The data is available upon reasonable reguest.
